# Isolated renal metastasis from squamous cell lung cancer

**DOI:** 10.1186/2049-6958-8-2

**Published:** 2013-01-16

**Authors:** Jun Cai, Gai Liang, Zhiqiang Cai, Ting Yang, Shuang Li, Jiyuan Yang

**Affiliations:** 1 Department of Oncology, First Affiliated Hospital of Yangtz University, Hubei, Jingzhou 434000, P.R. China

**Keywords:** Hematuria, Isolated unilateral renal metastases, Lung squamous cell carcinoma, Renal metastasis

## Abstract

Renal metastasis from non-small cell lung cancer is rather uncommon. The mechanism underlying the occurrence of metastasis in this site is still not well understood. We report a case of a 53-year-old Chinese woman who had moderately differentiated squamous cell carcinoma of the lung. After a ten months post-surgery interval of disease free survival, computed tomography (CT) scan found that left renal parenchymal was occupied by a mass, confirmed by kidney biopsy to be a metastasis from squamous cell lung carcinoma. Based on this case, we are warned to be cautious in diagnosis and treatment when renal lesion are detected.

## Background

The morbidity and mortality of lung cancer tend to increase at present, and its mortality rate is the highest among all types of cancer in China [1]. Although a great progress has been made in the treatments of this tumor, especially in non-small cell lung cancer (NSCLC), the 5 years survival rate of NSCLC is still lower than 15% [2]. More than 1/3 of NSCLC patients are diagnosed with distant metastasis, which is mainly due to difficulties in early detection. Local symptoms caused by primary tumors and complications due to distant metastases of NSCLC are major factors endangering patients’ lives. The most common site of metastasis via the hematogenous route is brain, followed by bone, liver, adrenal gland, and lung. However, metastasis of renal parenchymal is rather uncommon. Here we describe a case of lung carcinoma causing isolated unilateral renal metastases.

## Case presentation

**Figure 1:**
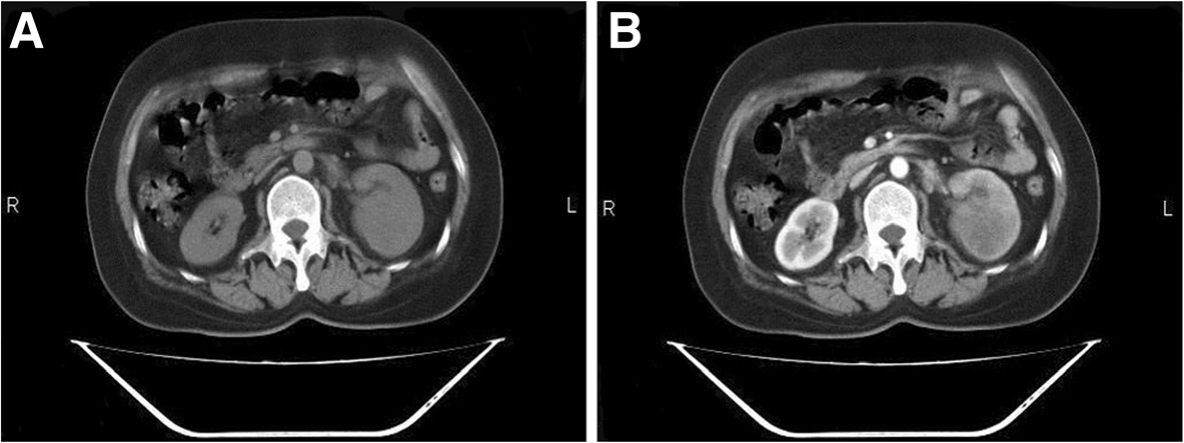
**NSCLC renal metastasis. **Computed tomography scan (**A**) and enhanced scan (**B**).

**Figure 2:**
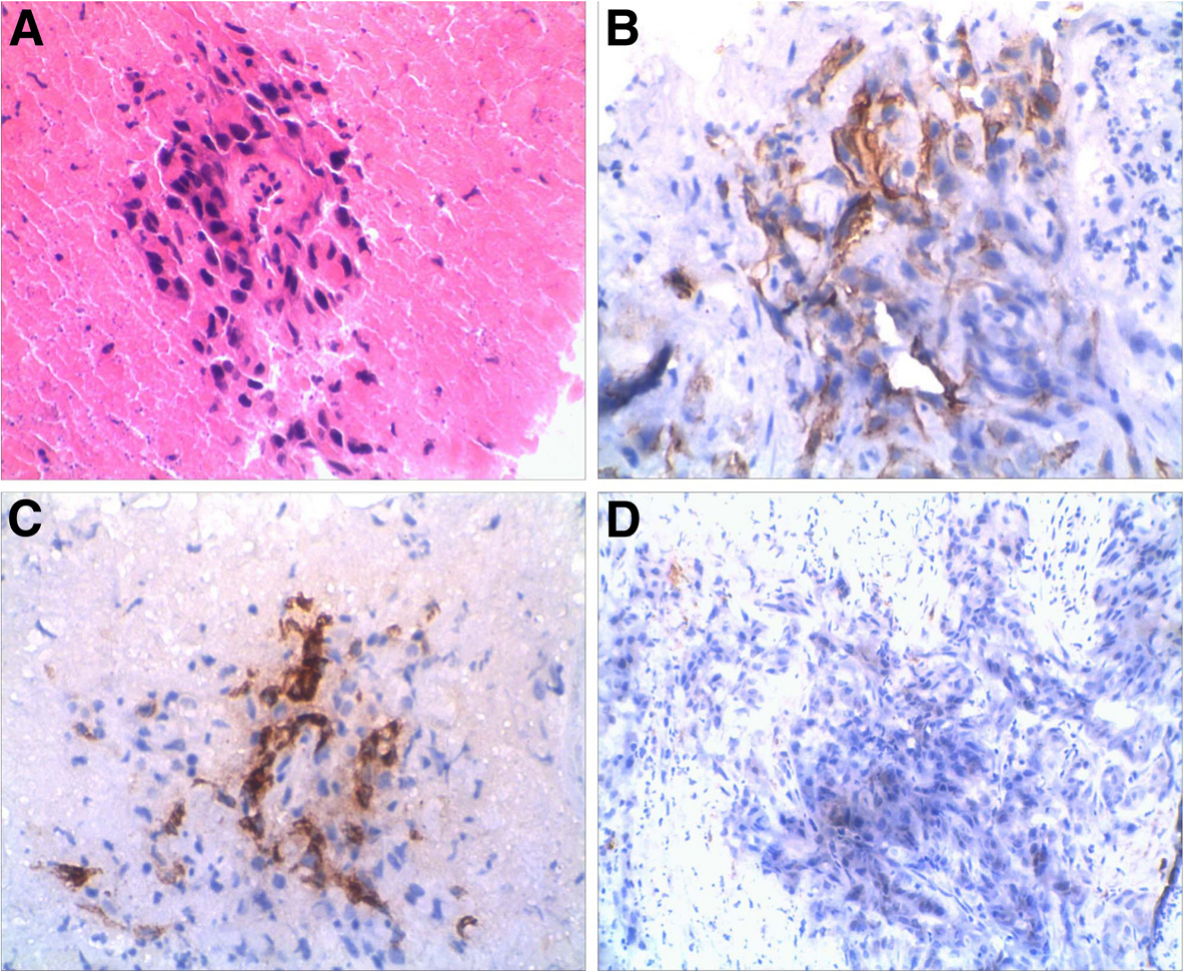
**NSCLC renal metastasis. **Renal biopsy specimen : Hematoxylin-eosin staining (**A**) and immunohistochemical staining of 34βE12 (**B**), CK5/6 (**C**) and TTF-1(**D**).

A 53-year-old Chinese woman presented symptoms of cough, bloody sputum at the first visit. One month later, enhanced chest CT scan showed a peripheral mass 5 cm in the greatest size in the left upper lung, without obvious enlargement of hilar and mediastinal lymph nodes. Subsequent whole body CT scan showed no distant metastasis in brain, liver and kidney. Then she had left upper lobectomy and chest lymph nodes exploration. Post-operation pathologic examination revealed moderately differentiated squamous cell carcinoma, with one metastatic lymph node in the fifth station, and two metastatic lymph nodes in the lung hilum (pT2N2M0). The immunohistochemical staining was positive for PCK, CK5/6, 34βE12, P63, CK17, and negative for CK19, TTF-1, CK7, CK20, Ki67 > 10%. After surgery, the patient was given four cycles of postoperative adjuvant chemotherapy with cisplatin and vinblastine. Because of incomplete dissection of mediastinal lymph nodes and pathologic N2 disease, the patient was subsequently treated with mediastinal radiotherapy, 50 Gy in all at 2 Gy per day, five days per week for five weeks. After 10 months of disease free interval, a routine urine test showed WBC 20 and RBC 5–10 per high-power field; enhanced CT scan showed an occupying lesion in the left renal parenchyma and paraaortic lymph node enlargement (Figure 1), while brain MRI and liver CT scan showed no other distant metastasis. In addition, B ultrasound guided left kidney biopsy was performed and immunohistochemical staining of the biopsy specimen showed: metastatic squamous cell lung carcinoma, with the same positive tumor biomarkers as in the surgical lung sample (Figure 2).

## Discussion

Tumor metastasis is a process that includes shedding, adhesion, penetration, migration and proliferation of primary tumor cells. A variety of cytokines, proteins, and genes are involved in the process. The tumor cells entering circulation do not spread to every organ randomly, but tend to seed in selected organs. Mechanisms underlying this targeted metastases are still unclear. The “seed soil” hypothesis proposed by Fidler [3] considers that the tumor cells transfer to any location equally, but they can only grow in specific sites able to provide suitable environment for the growth (soil). The tumor cells entering the cycle have receptors specifically binding to the endothelial cells of target organs through special chemotactic factors produced by them (seed); thus the tumor cells are preferentially attracted to the target organ (seed soil). In addition, the anatomical structure of the primary lesion, the inherent anatomic structure of the target organ and other factors can have effect on tumor production of metastates to target organs.

Renal metastases frequently do not present clinical symptoms and many patients have no hematuria or hyperazotemia. According to literature, renal metastatic tumors mainly locate in the cortical zone close to glomerular vascular plexus and seldom spread to urothelial tissue, so that the incidence of microscopic hematuria is 12% ~ 31% [4]. Single renal tumors include renal cell carcinoma and renal angiomyolipoma. Multiple renal tumors include multiple renal infarctions and multiple renal cysts. Identification of tumors metastatic to kidney, so as of renal cell carcinoma, renal angiomyolipoma and renal cysts, relies on kidney biopsy. In this report, the immunohistochemical staining of left kidney biopsy specimen showed positive CK5/6,34βE12, and P63 markers, whereas the TTF-1 was negative. These results indicated that the renal mass derived from the primary lung squamous cell carcinoma [5], and it was not a renal cell carcinoma. Renal metastases from NSCLC are restricted to a few anecdotal case reports in literature [6,7]. Even when present, renal metastases are usually part of disseminated disease or bilateral renal metastases [8], but in our patient the metastasis was single.

As to the treatment, it has to be individualized [9]: nephrectomy may be considered in the absence of disseminated disease or in selected patients with need for palliation. Our patient refused nephrectomy, so we treated her with Stereotactic Radiosurgery - Gama Knife for the left kidney. After radiotherapy, the patient remained under clinical observation.

## Conclusions

Renal metastatic tumors from squamous cell lung carcinoma are very rare and easy to be neglected, however we should take into account also the possibility of a metastasis from this tumor when a renal mass is detected.

## Consent

Written informed consent was obtained from the patient for publication of this case report and any accompanying images. A copy of the written consent is available for review by the Editor-in-Chief of this journal.

## Competing interests

The authors declare that they have no competing interests.
